# Changes in Thyrotropin Receptor Antibody Levels Following Total Thyroidectomy or Radioiodine Therapy in Patients with Refractory Graves' Disease

**DOI:** 10.1089/thy.2020.0756

**Published:** 2021-08-03

**Authors:** Jinyoung Kim, Min Sun Choi, Jun Park, Hyunju Park, Hye Won Jang, Jun-Ho Choe, Jung-Han Kim, Jee Soo Kim, Young Seok Cho, Joon Young Choi, Tae Hyuk Kim, Jae Hoon Chung, Sun Wook Kim

**Affiliations:** ^1^ Division of Endocrinology and Metabolism, Department of Medicine, Thyroid Center, Samsung Medical Center, Sungkyunkwan University School of Medicine, Seoul, Korea.; ^2^ Department of Medical Education, Sungkyunkwan University School of Medicine, Seoul, Korea.; ^3^ Division of Endocrine Surgery, Department of Surgery, Samsung Medical Center, Sungkyunkwan University School of Medicine, Seoul, Korea.; ^4^ Department of Nuclear Medicine, Samsung Medical Center, Sungkyunkwan University School of Medicine, Seoul, Korea.

**Keywords:** Graves'disease, radioactive iodine therapy, thyrotropin receptor antibody, thyrotropin-binding inhibitory immunoglobulins, total thyroidectomy

## Abstract

***Background:*** The actions of thyrotropin-binding inhibitory immunoglobulins (TBIIs) against thyrotropin receptors in thyroid follicular cells have been studied as important etiological factors in Graves' disease (GD). The purpose of this study was to investigate changes in the TBII levels of patients undergoing total thyroidectomy (TTx) or radioactive iodine (RAI) therapy for GD refractory to antithyroid drugs (ATDs).

***Methods:*** We enrolled patients who underwent TTx or RAI for GD with previous ATD use between January 2011 and December 2017 at the Samsung Medical Center in Seoul, Korea. Thorough retrospective reviews of medical records were performed in 130 patients.

***Results:*** Patients with goiter, ophthalmopathy, high levels of TBIIs, and high doses of ATDs received TTx. Elderly patients with arrhythmia received RAI. We observed that TBII levels continued to decrease after TTx. On the contrary, TBIIs initially increased for 138 days (estimated median time) and then decreased slowly after RAI. A faster decline in TBII levels was observed in the TTx group than in the RAI group (*p* < 0.001). The estimated median time for TBIIs to decrease below 4.5 IU (3 × upper normal limit, which is known to be a risk factor for fetal hyperthyroidism) was 318 days in the TTx group and 659 days in the RAI group, respectively. In the RAI group, high levels of TBII (>4.5 IU/L) were present in 70 (82%) at 6 months, 57 (67%) at 1 year, and 3 (3%) at 2 years. In the TTx group, rapid decreases in TBII levels were observed in younger patients and those with lower baseline TBII levels. In the RAI group, smaller thyroid volume was correlated with more rapid decrease in TBII levels.

***Conclusions:*** The changes in TBII levels following TTx or RAI were different in patients with refractory GD. When deciding on TTx or RAI, this difference should be considered with patient age, severity of hyperthyroidism, goiter, ophthalmopathy, and future pregnancy plans (for young female patients).

## Introduction

Serum thyrotropin (TSH) receptor antibodies have been demonstrated to be an important etiology factor for Graves' disease (GD) (1). These antibodies are thought to promote the function and growth of thyroid follicular cells, leading to hyperthyroidism and hypertrophy of the thyroid gland. Thyroid-stimulating antibodies can be measured in a number of ways but, to date, the method preferred for clinical convenience is the thyrotropin-binding inhibitory immunoglobulin (TBII) assay (2).

TBII levels have high sensitivity and specificity in the diagnosis of GD (3). Although controversial (4), they also help diagnose ophthalmopathy accompanying GD (5) and predict the clinical course of eye disease (6). In pregnant GD patients, maternal TBII levels in the latter half of pregnancy are associated with fetal and neonatal hyperthyroidism (7). Therefore, measurement of TBII levels is recommended in pregnant GD patients (8).

Antithyroid drugs (ATDs), total thyroidectomy (TTx), and radioactive iodine (RAI) therapy are current treatment options for GD (9). ATDs are thionamide-based chemicals that not only inhibit the synthesis of thyroid hormones but also have immunosuppressive effects, which can be confirmed by decreases in TBII levels during the treatment course (10). TBII levels at the start of ATD therapy, the rate of decrease in TBII levels during ATD treatment, and TBII levels at the time of treatment cessation can predict relapse (11–13).

TTx involves removal of the thyroid, and RAI destroys the thyroid because ionizing radiation causes DNA damage. Since TTx is anatomically invasive and RAI is accompanied by exposure to radiation, both are considered more aggressive treatments than ATDs. Furthermore, in addition to their invasiveness, the requirement for lifelong thyroid hormone replacement therapy after RAI and TTx leads to a preference for ATDs as a primary therapy in Asia and Europe (14,15).

Although ATDs are the preferred treatment, TTx or RAI is required as a definitive treatment when faced with the progression or relapse of GD, especially as more than half of the patients suffer relapse after the cessation of ATDs (16). Patients in this study chose TTx or RAI over long-term ATD use after physicians explained to them their treatment options for fluctuating symptoms. In these cases, changes in TBII levels could have important implications as indicators of immune response, especially in patients with ophthalmopathy and in patients planning for pregnancy. The purpose of this study was to understand changes in TBII levels in patients undergoing TTx or RAI as a definitive treatment for GD not successfully treated by a previous course of ATDs.

## Methods

### Patients

**FIG. 1.**
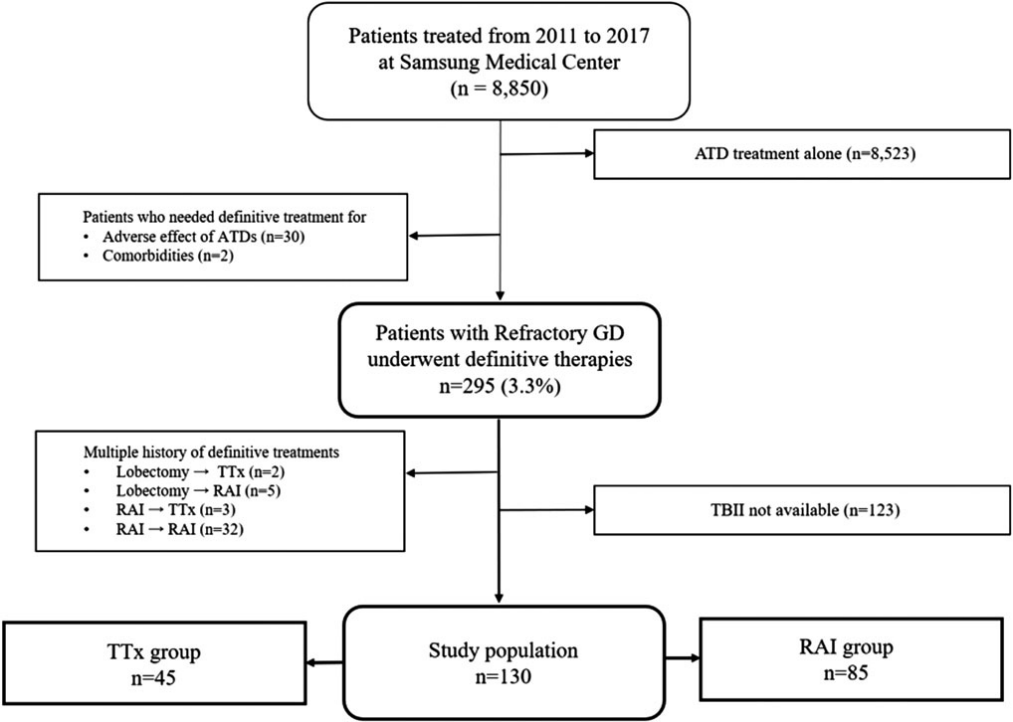
Flowchart of the study population.

After receiving Institutional Review Board approval (SMC 2020-05-184), we retrospectively reviewed patients who underwent TTx or RAI between 2011 and 2017 at the Samsung Medical Center, Seoul, Korea. The follow-up time continued through 2019. We enrolled 130 patients who required definitive treatment even after sufficient ATD use, and for whom TBII levels were available 3 months before treatment and at least once within 1 year after treatment (Fig. 1). The median number of TBII measurements following definitive treatment during the 2-year study period was 3, with a range of 1–8.

The exclusion criteria were as follows: patients who received TTx or RAI due to the initial side effects of ATDs (Supplementary Table S1) or who had other comorbidities, including one patient with active hepatitis in the RAI group and one patient with a large thyroid nodule in the TTx group; patients with histories of thyroid lobectomy before TTx or RAI; patients who underwent TTx after RAI failure (Supplementary Table S2); and patients who received multiple courses of RAI during the study period (Supplementary Table S3).

### Biochemical analysis and clinical assessment

Serum free thyroxine, total triiodothyronine (T3), and TSH concentrations were measured using a radioimmunoassay method (Beckman Coulter, La Brea, CA) with the following reference ranges: 0.64–1.72 ng/dL, 76–190 ng/dL, and 0.3–6.5 mU/L, respectively. Hyperthyroidism was defined as a TSH level of less than or equal to 0.05 mU/L (also for recurrence of hyperthyroidism after RAI). TBII levels were measured using a second-generation TRAK human assay (Thermo Fisher Scientific, Waltham, MA) and values greater than 1.5 U/L were regarded as positive results.

Thyroid volume was examined in all patients by the treating physician at the time of treatment and classified according to the World Health Organization standard (i.e., grade 0, no goiter; grade 1, palpable goiter; or grade 2, visible goiter in the normal position of the neck). Patients who had Graves' ophthalmopathy (GO) were defined as those who had been diagnosed with thyroid eye disease before TTx or RAI treatment. Patients who had arrhythmia were defined as those who had been diagnosed with clinical arrhythmias other than sinus tachycardia that required medical intervention.

### Treatments

For patients receiving TTx, surgery was performed by experienced surgeons at our institution. For patients in the RAI group, the dose of liquid radioiodine to be administered was decided based on the fixed-activity approach at the treating physicians' discretion. Patients were instructed to maintain an iodine-restricted diet and stop ATDs for five to seven days before receiving treatment. Glucocorticoid was prescribed for preventive purposes in patients with ophthalmopathy to prevent exacerbation of thyroid eye diseases.

### Statistical analyses

The continuous variables are presented as mean ± standard deviation and differences were compared using the Mann–Whitney *U* test. Quantitative variables are summarized as the count (the percentage of the total) and analyzed using Fisher's exact test.

We used survival analysis for TBII normalization, and the event was defined as the level of TBII less than 4.5 IU (3 × the upper normal limit, which is known to be a risk factor for fetal hyperthyroidism). Calculations of TBII reduction period and comparisons between groups were assessed using the Kaplan–Meier method and log-rank test. To analyze factors influencing changes in TBII levels, univariable and multivariable Cox regression models were used. For these categorical variables in the Cox analysis, multiple comparison with Bonferroni's method was used to correct the *p*-value and confidence interval.

Statistical analyses were performed using R version 3.6.3. (R Foundation for Statistical Computing, Vienna, Austria) and SAS version 9.4 (SAS Institute, Cary, NC).

## Results

### Patient characteristics

Patients in the TTx group had higher T3 (*p* = 0.009) and TBII (*p* < 0.001) levels. More patients had goiter (*p* < 0.001) and ophthalmopathy (*p* < 0.001) in the TTx group than in the RAI group. Meanwhile, the patients in the RAI group were older (*p* = 0.006) and there were more patients with arrhythmia (*p* = 0.012) in the RAI group than in the TTx group (Table 1).

**Table 1.** Characteristics of Graves' Patients at the Time of Treatment

	Total thyroidectomy (*n* = 45)	Radioiodine therapy (*n* = 85)	*p*
Age, years, mean ± SD	40.24 ± 11.33	47.06 ± 14.17	0.006
Sex, male, *n* (%)	9 (20.0)	30 (35.3)	0.108
Height, cm, mean ± SD	161.81 ± 7.14	162.88 ± 9.28	0.501
Weight, kg, mean ± SD	63.71 ± 10.81	61.94 ± 13.24	0.444
Number of relapses	0.42 ± 0.84	0.49 ± 0.88	0.654
TSH, μIU/mL, mean ± SD	0.23 ± 0.80	0.17 ± 0.88	0.714
T3, ng/dL, mean ± SD	197.82 ± 111.09	158.98 ± 54.71	0.009
Free T4, ng/dL, mean ± SD	1.58 ± 0.77	1.78 ± 0.69	0.143
TBII, IU/L, mean ± SD	66.86 ± 87.77	21.27 ± 48.25	<0.001
Goiter, WHO classification, *n* (%)			<0.001
Grade 0	4 (8.9)	41 (48.2)	
Grade 1	8 (17.8)	27 (31.8)	
Grade 2	33 (72.3)	17 (20.0)	
Ophthalmopathy, *n* (%)	25 (55.6)	17 (20.0)	<0.001
Arrhythmia, *n* (%)	2 (4.4)	20 (23.5)	0.012
ATD, type, *n* (%)			0.094
Methimazole	36 (80.0)	59 (69.4)	
Carbimazole	8 (17.8)	17 (20.0)	
Propylthiouracil	1 (2.2)	9 (10.6)	
ATD dose,^a^ mg/day, mean ± SD	23.28 ± 13.58	18.38 ± 12.93	0.046
ATD use, years, mean ± SD	3.92 ± 3.56	4.24 ± 3.25	0.411

^a^ The dose of ATDs at the time of definitive treatment. Doses were converted based on methimazole (methimazole:carbimazole:propylthiouracil = 1:0.6:10).

ATD, antithyroid drug; SD, standard deviation; TBII, thyroid binding inhibitory immunoglobulin; TSH, thyroid-stimulating hormone.

All patients had been prescribed ATDs as first-line therapy. The average duration of medical treatment was 4.13 years before undergoing definitive treatment. The most common type of ATD prescribed was methimazole (73%), while some patients were treated with carbimazole (19%) or propylthiouracil (8%). There were no significant differences in the duration of treatment or type of ATD used between the RAI and TTx groups (*p* = 0.094). However, the dose of ATD prescribed to control hyperthyroidism at the time of treatment was significantly higher in the TTx group (*p* = 0.046) (Table 1).

### Clinical outcomes

All patients in the TTx group (100%) reached hypothyroidism immediately after the treatment. On the contrary, 35 patients (41.2%) who had undergone RAI had recurrent hyperthyroidism and 16 patients (18.8%) did not experience remission during the 2-year study period.

Adverse effects were observed in 2 of the 130 patients (1.5%). After TTx, one patient complained of voice change, but the issue improved within six months but vocal cord palsy was not confirmed. One patient who did not receive steroid prophylaxis was newly diagnosed with GO three months after RAI.

### Changes in TBII

**FIG. 2.**
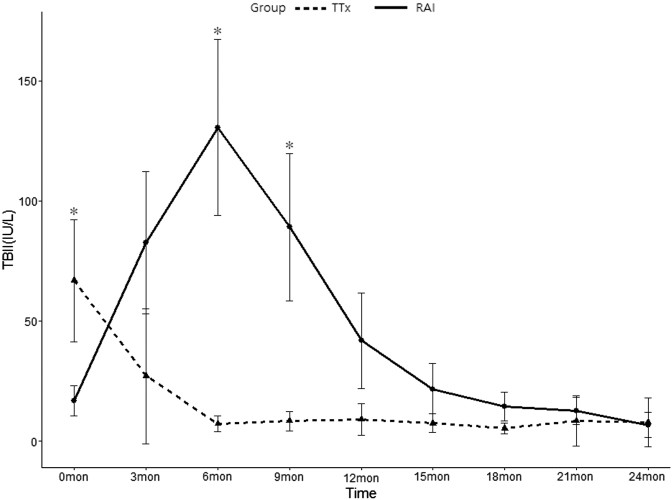
TBII levels over 24 months after radioiodine treatment or TTx. Adjusted means and confidence intervals by generalized estimating equation for repeated measures analysis are shown. Tukey's multiple comparison tests are presented as * for significance levels of *p* < 0.05. TBII, thyrotropin-binding inhibitory immunoglobulin; TTx, total thyroidectomy.

Changes in TBII levels over a period of two years after RAI or TTx were analyzed. TBII levels continued to decrease in the TTx group following treatment, while, conversely, TBII levels increased for 138 days (estimated median value) and decreased slowly thereafter in the RAI group. There were statistically significant differences in TBII levels between the two groups at six and nine months after treatment (Fig. 2).

**FIG. 3.**
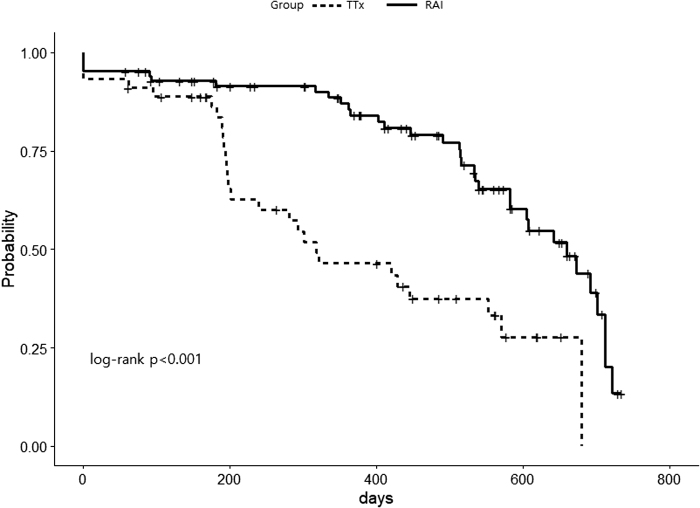
Probability of persistent high TBII levels (>4.5 IU/L) over the 24 months following TTx or RAI treatment. RAI, radioactive iodine.

We analyzed various factors affecting decreases in TBII levels. The most important factor in those patients achieving immunologic remission appeared to be the treatment modality. Although patients in the TTx group had more severe disease, higher TBII levels, and goiter, they had a faster decline in TBII (*p* < 0.001) than those in the RAI group. The estimated median times for TBII to decrease below 4.5 IU (upper normal limit 3 × ) were 318 days in the TTx group and 659 days in the RAI group (Fig. 3).

Since the pattern of changes in TBII levels varied according to the treatment method, factors affecting the reduction in TBII levels were analyzed by subgroup analysis in each treatment group. In the TTx group, older patients (*p* < 0.01) and those with high TBII levels (*p* < 0.05) before undergoing treatment were found to have slower reductions in TBII levels. In the RAI group, goiter (*p* < 0.05) was associated with a longer time for the TBII levels to decrease (Table 2). The duration of prior ATD was not associated with TBII reduction in either group.

**Table 2.** Cox Regression for Thyrotropin-Binding Inhibitory Immunoglobulin Reduction

Group	Total thyroidectomy			Radioiodine therapy		
	HR	[CI]	*p*	HR	[CI]	*p*
**Univariable analysis**						
Age, years	0.95	[0.91–0.98]	<0.01	1.00	[0.98–1.03]	0.77
Goiter^a^						
Grade 0 (reference)	1.00			1.00		
Grade 1	0.74	[0.11–4.90]	>0.99	0.69	[0.30–1.60]	0.65
Grade 2	0.72	[0.13–3.87]	>0.99	0.10	[0.01–0.94]	0.04
Quartiles of pretreatment TBII^a^						
Q1 (reference)	1.00			1.00		
Q2	0.26	[0.07–0.99]	0.05	1.27	[0.41–3.96]	>0.99
Q3	0.16	[0.04–0.68]	<0.01	1.33	[0.45–3.94]	>0.99
Q4	0.32	[0.10–1.09]	0.08	0.23	[0.04–1.48]	0.17
Quartiles of the ATD duration^a^						
Q1 (reference)	1.00			1.00		
Q2	0.66	[0.15–2.83]	>0.99	1.14	[0.42–3.12]	>0.99
Q3	1.00	[0.26–3.86]	>0.99	0.54	[0.15–1.94]	0.74
Q4	0.76	[0.21–2.80]	>0.99	0.51	[0.15–1.71]	0.54
**Multivariable analysis**						
Age, years	0.94	[0.89–0.98]	<0.01	0.99	[0.96–1.02]	0.50
Goiter^a^						
Grade 0 (reference)	1.00			1.00		
Grade 1	0.37	[0.03–4.07]	0.71	0.64	[0.23–1.77]	0.65
Grade 2	0.56	[0.05–6.29]	>0.99	0.12	[0.01–1.44]	0.11
Quartiles of pretreatment TBII^a^						
Q1 (reference)	1.00			1.00		
Q2	0.20	[0.04–0.90]	0.03	0.20	[1.36–3.96]	>0.99
Q3	0.15	[0.03–0.85]	0.03	1.25	[0.40–3.90]	>0.99
Q4	0.19	[0.04–0.87]	0.03	0.33	[0.05–2.29]	0.52
Quartiles of the ATD duration^a^						
Q1 (reference)	1.00			1.00		
Q2	0.73	0.12–4.59	>0.99	1.36	[0.48–3.83]	>0.99
Q3	0.99	0.13–7.36	>0.99	1.01	[0.23–4.40]	>0.99
Q4	0.95	0.18–4.90	>0.99	0.59	[0.17–2.08]	0.94

^a^ For these categorical variables, multiple comparison with Bonferroni's method was used to correct the *p*-value and confidence interval.

CI, confidence interval; HR, hazard ratio; Q, quartile group of continuous variables.

## Discussion

We studied changes in TBII levels after TTx or RAI as definitive treatment of GD refractory to ATD therapy. During the 7-year study period, a total of 8850 patients were treated for GD at our thyroid center. ATD was administered as an initial treatment for all patients. The number of patients who had TTx or RAI for GD was 327 (3.69%), 85 and 242, respectively. TTx was recommended in patients with severe eye symptoms or large goiters, and RAI was recommended in elderly patients at high cardiovascular risk. As expected, the TTx patient group had more GO and the RAI group patients were older and were more often treated for arrhythmia (Table 1).

There are clinical cases in which TTx or RAI is required for nonrefractory GD, due to the occurrence of ATD-related side effects. In previous studies, the prevalence of minor side effects such as cutaneous reactions, arthralgia, and gastrointestinal symptoms was reported to be 5%, and more serious side effects such as hepatitis or agranulocytosis were reported in 0.3–0.4% of patients (17,18). During the study period, serious adverse effects were observed in a total of 30 patients (0.34%) on ATDs, and 4 and 26 patients who had TTx and RAI, respectively. The adverse events observed in the current study were generalized rash, arthralgia, hepatitis, and agranulocytosis (Supplementary Table S1).

In our study cohort, we found that patients in the TTx group exhibited faster decline in TBII levels, consistent with the results of a previous study (19). We also confirmed that hyperthyroidism was immediately resolved after TTx. The reduction in TBII levels in the TTx group occurred faster, despite these individuals having more severe symptoms before treatment. TTx was selected as the definitive treatment in patients with severe GD who were not responsive to ATDs, as well as in patients who could not be treated with RAI (Supplementary Table S2). From this point of view, TTx is the most reliable option in terms of definitive treatment for hyperthyroidism due to GD.

Even though the effects of TTx are immediate and the adverse events are few in number (20), it remains difficult to recommend TTx as an initial treatment when considering its cost and invasiveness. The results of this study indicate that patients with severe GD, who had high TBII levels, goiter, and ophthalmopathy, often underwent TTx. In elderly patients or patients with arrhythmia, RAI would be preferred over surgery and these patients would likely easily accept RAI as the definitive treatment option.

RAI is often chosen as the first-line treatment for GD in some centers because it has a higher success rate than ATD therapy (21,22). In Western countries, the success rate of RAI has been reported to be as high as 90% (23). The success rate in the current study population was 81.2% for the first RAI. The success rate of RAI is thought to be lower in patients with refractory GD, and so, our results are not surprising considering that this study is limited to patients who were difficult to treat with ATDs.

The post-treatment deterioration of eye disease has been suggested as a possible adverse effect of RAI and was confirmed to occur at a rate of 7% in previous studies (24,25). Existing guidelines recommend that oral prednisolone prophylaxis be given to patients at high risk (26). At our institution, glucocorticoid was administered to patients with GO for a period of two to four weeks depending on the patient's risk stratification. Among the patients studied, one patient (1.2%) reported newly developed ophthalmic symptoms at three months after RAI.

Due to the radiation exposure inherent in the treatment and the fact that TBII levels remain high for a long period of time after RAI, this therapeutic approach may be less desirable in women of childbearing age. Neonatal thyrotoxicosis has been reported in pregnant women after RAI (27). In this study, we tried to pinpoint the time when TBII falls below 4.5 IU/L, because it is known that the probability of fetal hyperthyroidism is significantly increased at a level three times that of the TBII upper normal value (1.5 IU/L) (7,8,28,29). With RAI, the estimated median time for high-level TBII (>4.5 IU/L) was 659 days, and the numbers at risk of high-level TBII (>4.5 IU/L) were 70 (82%) at 6 months, 57 (67%) at 1 year, and 3 (3%) at 2 years (Fig. 2). These results show that the time to normalization of TBII levels is variable, and that it could take more than two years in some patients. Therefore, we suggest that clinicians discuss treatment options with patients during counseling if the patients intend to get pregnant, even if it is recommended to wait to plan pregnancy until six months after RAI due to the dangers associated with direct radiation exposure effects to a fetus (30).

During the study period, 32 patients received a second RAI (Supplementary Table S3) at a median of 1.2 years after the first RAI. When the second RAI group was further analyzed, the baseline TBII level was higher than that seen in the first RAI group, which may be due to the previous RAI treatment (17.55 ± 26.95 IU/L vs. 70.19 ± 90.43 IU/L, *p* < 0.001). Patients who failed to respond to the first RAI tended to have larger goiters than the first RAI-only group (*p* = 0.009), which is consistent with the tendency of patients who do not respond well to ATDs to have a large goiter (31). The number of patients at risk of high-level TBII (>4.5 IU/L) was 7 (22%) at 2 years. Detailed analysis was difficult because of the short period of follow-up, but immunologic remission defined as a decrease in TBII may take longer than two years in patients who have multiple RAI therapies.

Laurberg et al. (32) previously studied changes in TBII levels in patients with untreated Graves' hyperthyroidism following the three-most common therapies, ATDs, sub-TTx, and RAI. In this study, the ATD group reported earlier immunologic remission at six months than the surgical group, which required one year. ATD appears to be superior to surgery in terms of immunosuppressive effects, but it is worth noting that sub-TTx was selected as a surgical treatment in the study at the time, not TTx. TTx is now recommended as a standard method for surgical treatment of GD due to frequent recurrences after sub-TTx and the higher risk of complications for reoperation (33–35). In this study, even in refractory GD patients who were not controlled by ATD, definite decreases in TBII were confirmed. TTx is recommended for patients with severe symptoms requiring immediate improvement of hyperthyroidism, patients with a large goiter or severe thyroid eye diseases, and young women considering pregnancy in the near future.

We used the second-generation assay for TBII measurement since our hospital introduced it in 2009, unlike Laurberg et al.'s study (32) that used the first-generation assay. Although this study showed similar results to those of other studies using second-generation TBII levels (19,36), we additionally interpreted changes in TBII levels and applied the information gathered in the clinical settings. Moreover, we performed additional analyses and estimated the period of reduction. Factors that influenced actual TBII reduction were also analyzed. We believe that the early application of TTx in young patients not only improves hyperthyroidism rapidly but also helps to reduce TBII early. When RAI is administered to patients with large goiters, elevated TBII levels could last for a long time (Table 2). Considering the current clinical situation in which most patients received ATDs as their first line treatment, we assessed whether long-term use of ATD affects changes in TBII following TTx or RAI treatment. We found that the previous treatment period with ATDs was not associated with TBII levels.

This study is limited by its retrospective design and small sample size. There was no randomization process for the patient groups that received different treatments, and changes in TBII for ATD therapy were not covered in this study. The follow-up period was set to two years, which is shorter than the five-year period in a previous study (32) because patients were often referred to primary care providers after definitive treatment. Also, a selection bias cannot be excluded because the levels of TBII that the study sought to focus on were not measured in many patients whom we treated, and there were missing values of TBII levels in our study population.

In conclusion, changes in TBII levels after TTx or RAI were different in patients with GD refractory to ATDs. When deciding on TTx or RAI, this difference should be considered with patient age, severity of hyperthyroidism, goiter, ophthalmopathy, and future pregnancy plans (for young female patients).

## Acknowledgment

The authors thank the Statistics and Data Center at the Samsung Medical Center for statistical support.

## Authors' Contributions

Conception or design: S.W.K. and J.K. Acquisition, analysis, or interpretation of data: J.K., M.S.C., J.P., H.P., H.W.J., J.H.C., J.H.K., J.S.K., Y.S.C., J.Y.C., T.H.K., J.H.C., and S.W.K. Drafting the work or revising: J.K. and S.W.K. Final approval of the article: J.K. and S.W.K.

## Supplementary Material

Supplemental data

Supplemental data

Supplemental data
