# Supplementary material for: Changes in Thyrotropin Receptor Antibody Levels Following Total Thyroidectomy or Radioiodine Therapy in Patients with Refractory Graves' Disease
Source: Thyroid. 2021 Aug 3;31(8):1264–71. doi: 10.1089/thy.2020.0756 (PMC8377510; doi:10.1089/thy.2020.0756)
Supplement: Supplemental data [file Sppl_TableS1.docx]

**Supplementary Table S1.**

**Characteristics of Patients Reporting Drug Adverse Effects**

| *Characteristics* | n = 30 |
| --- | --- |
| Age at treatment, years (mean$\boldsymbol{\pm}$SD) | 43.30 $\pm$17.22 |
| Sex, men (%) | 6 (20%) |
| Goiter, WHO classification  Grade 0  Grade 1  Grade 2 | 15 (50%)  9 (30%)  6 (20%) |
| Ophthalmopathy | 8 (27%) |
| TBII, IU/L (mean$\boldsymbol{\pm}$SD) | 30.48$\pm41.4$ |
| TSH, μIU/mL (mean$\boldsymbol{\pm}$SD) | 0.02$\pm$0.02 |
| T3, ng/dL (mean$\boldsymbol{\pm}$SD) | 258.58$\pm$113.95 |
| Free T4, ng/dL (mean$\boldsymbol{\pm}$SD) | 2.76 $\pm$ 0.96 |
| ATD, type |  |
| Methimazole (%)  Propylthiouracil (%) | 21 (70%)  9 (30%) |
| ATD dose†, mg (mean$\boldsymbol{\pm}$SD) | 17.7 $\pm$ 9.9 |
| **ATD duration, days (mean**$\boldsymbol{\pm}$**SD)** | **54.4** $\boldsymbol{\pm}$ **56.6** |
| Types of adverse effects  Agranulocytosis  Generalized rash  Hepatitis  Severe arthralgia | 19 (63%)  3 (10%)  7 (23%)  1 (4%) |
| Definitive Treatment  RAI  TTx | 26 (87%)  4 (13%) |

TSH, thyroid stimulating hormone; TBII, thyroid binding inhibitory immunoglobulin ; ATD, antithyroid drug; RAI, radioactive iodine; TTx, total thyroidectomy †Doses were converted based on methimazole (Methimazole : Carbimazole : Propylthiouracil = 1 : 0.6 : 10)
