# Supplementary material for: Changes in Thyrotropin Receptor Antibody Levels Following Total Thyroidectomy or Radioiodine Therapy in Patients with Refractory Graves' Disease
Source: Thyroid. 2021 Aug 3;31(8):1264–71. doi: 10.1089/thy.2020.0756 (PMC8377510; doi:10.1089/thy.2020.0756)
Supplement: Supplemental data [file Sppl_TableS2.docx]

**Supplementary Table S2.**

**Patients who Underwent Total Thyroidectomy after Radioiodine Therapy Failure**

| Age | Sex | Goiter | TBII | TSH | T3 | Free T4 | History of Radioiodine Therapy |
| --- | --- | --- | --- | --- | --- | --- | --- |
| years |  | grade | μIU/mL | ng/dL | ng/dL | ng/dL |  |
| 27 | F | grade 2 | 12.9 | 0.01 | 562.5 | 5.32 | Once a month before surgery  Surgery was performed due to worsening hyperthyroidism after radioiodine therapy. |
| 23 | F | grade 2 | 400.0 | 3.21 | 102.1 | 0.12 | Once a year before surgery  Surgery was performed after the first relapse. |
| 47 | F | grade 2 | 580.0 | 0.07 | 322.5 | 1.95 | 3 times 5 years before surgery  Surgery was performed after the fourth relapse. |

TBII, thyroid binding inhibitory immunoglobulin; TSH, thyroid stimulating hormone
