# Supplementary material for: Changes in Thyrotropin Receptor Antibody Levels Following Total Thyroidectomy or Radioiodine Therapy in Patients with Refractory Graves' Disease
Source: Thyroid. 2021 Aug 3;31(8):1264–71. doi: 10.1089/thy.2020.0756 (PMC8377510; doi:10.1089/thy.2020.0756)
Supplement: Supplemental data [file Sppl_TableS3.docx]

**Supplementary Table S3.**

**Characteristics of Patients Receiving Second Radioiodine Therapy**

| *Characteristics* | n = 32 |
| --- | --- |
| Age at treatment, years (mean$\boldsymbol{\pm}$SD) | 39.22$\pm12.92$ |
| Sex, male (%) | 14 (43.8%) |
| **Goiter, WHO classification**  **Grade 0**  **Grade 1**  **Grade 2** | **5 (15.6%)**  **13 (40.6%)**  **14 (43.8%)** |
| Ophthalmopathy | 2 (6.2%) |
| **TBII, IU/L (mean**$\boldsymbol{\pm}$**SD)** | **70.19**$\boldsymbol{\pm}$ **90.43** |
| TSH, μIU/mL (mean$\boldsymbol{\pm}$SD) | 0.05$\pm$ 0.13 |
| T3, ng/dL (mean$\boldsymbol{\pm}$SD) | 180.73$\pm$ 104.73 |
| Free T4, ng/dL (mean$\boldsymbol{\pm}$SD) | 2.02$\pm$ 0.99 |
| ATD, type |  |
| Methimazole (%)  Carbimazole (%)  Propylthiouracil (%) | 26 (81.3%)  2 (6.2%)  4 (12.5%) |
| ATD dose†, mg (mean$\boldsymbol{\pm}$SD) | 18.92$\pm$ 9.86 |
| ATD duration, years (mean$\boldsymbol{\pm}$SD) | 5.47$\pm$ 5.64 |

TSH, thyroid stimulating hormone; TBII, thyroid binding inhibitory immunoglobulin ; ATD, antithyroid drug; RAI, radioactive iodine; TTx, total thyroidectomy †Doses were converted based on methimazole (Methimazole : Carbimazole : Propylthiouracil = 1 : 0.6 : 10)
